# Improved long-term survival with subdural drains following evacuation of chronic subdural haematoma

**DOI:** 10.1007/s00701-017-3095-2

**Published:** 2017-03-27

**Authors:** Mathew R. Guilfoyle, Peter J. A. Hutchinson, Thomas Santarius

**Affiliations:** Division of Neurosurgery, Addenbrooke’s Hospital, Box 167, Cambridge Biomedical Campus, Cambridge, CB2 0QQ UK

**Keywords:** Chronic subdural haematoma, Survival, Drain, Randomised controlled trial

## Abstract

**Background:**

Chronic subdural haematoma (CSDH) is a common condition that is effectively managed by burrhole drainage but requires repeat surgery in a significant minority of patients. The Cambridge Chronic Subdural Haematoma Trial (CCSHT) was a randomised controlled study that showed placement of subdural drains for 48 h following burrhole evacuation significantly reduces the incidence of reoperation and improves survival at 6 months. The present study examined the long-term survival of the patients in the trial.

**Methods:**

In the original trial patients at a single neurosurgical centre from 2004–2007 were randomly assigned to receive a drain (n = 108) or no drain (n = 107) following burrhole drainage of CSDH. We ascertained whether the trial patients were alive in February 2016—a minimum of 8 years following enrollment—via the UK NHS tracing service. Survival was compared between the trial groups and against expected survival for the UK general population matched for age and sex.

**Results:**

At 5 years following surgery the drain group continued to have significantly better survival than the no drain patients (p = 0.027), but this was no longer apparent at 10 years. Survival of patients in the drain group did not differ significantly from that of the general population whereas patients who did not receive a drain had significantly lower survival than expected (p = 0.0006).

**Conclusion:**

Subdural drains following CSDH evacuation are associated with improved long-term survival, which appears similar to that expected for the general population of the same age and sex. All patients having burrhole CSDH evacuation should receive a drain as standard practice unless specifically contraindicated.

## Introduction

Chronic subdural haematoma (CSDH) is a common condition, predominantly affecting those over 65 years of age [4]. Symptomatic CSDH is typically managed by surgical evacuation, usually via burrholes or mini-craniotomy, and a significant minority of patients develop recurrent symptomatic collections that require repeat surgical drainage.

From November 2004 to December 2007 the Cambridge Chronic Subdural Haematoma (CCSDH) trial randomised patients to receive a subdural drain for 48 h post-operatively (n = 108) or no drain (n = 107), following burrhole evacuation of CSDH at a single UK neurosurgical centre [7]. The incidence of recurrent CSDH necessitating redrainage was significantly lower in the patients who received a drain (9.3% vs. 24%, p = 0.003) and 6-month mortality was also significantly reduced (8.6% vs. 18.1%, p = 0.042) [7].

Although the prognostic implications of various clinical features and management factors have been reported in the literature [5], it remains unknown whether treated CSDH has an impact on patients’ long-term life expectancy. Patients with CSDH are generally from an age group with higher baseline mortality and it is difficult to infer from unadjusted data whether there is an excess of deaths following CSDH or if the observed survival curves are comparable to those of the age- and sex-matched general population.

The objective of this report is to detail the long-term survival of patients enrolled in the CCSDH trial, with a minimum 8-year follow-up, and to examine the relative survival of the study patients in comparison with the UK general population.

## Methods

The trial design and protocol, together with detailed demographic and clinical details of the trial participants, can be found in the original publication [7]. Briefly, adult patients requiring evacuation of CSDH were randomly allocated to receive either a subdural drain or no drain at the end of the burrhole drainage procedure. Drains were left on free drainage for 48 h prior to removal on the ward. The primary outcome measure was symptomatic recurrent CSDH needing repeat surgery; secondary outcomes included mortality and neurological outcome at 6 months following surgery. The trial was prospectively registered (ISRCTN 97314294) and approved by the UK NHS Research Ethics Committee. No funding was received for the study.

The survival status of trial patients was ascertained on 1 February 2016 via the UK NHS Strategic Tracing Service. This was 8 years from the date of enrollment of the last patient into the trial.

All statistical analysis was performed in R (v.3.2.4, www.r-project.org) using the *survival* and *relsurv* packages [6, 8] (both available at cran.r-project.org). Survival of the trial patients was calculated using the Kaplan-Meier method from the date of surgery. Survival between the study arms was compared with the two-sample log-rank test.

Life tables for the England and Wales civilian population, reporting the annual age- (in 1-year increments) and sex-specific risk of death for the years 1841–2013, were obtained from the Human Mortality Database (www.mortality.org). Data for 2013 were used for all later years as this was the most recent available. Life tables were used to generate representative cohorts of the general population with the same number of patients and identical age and sex profiles as the control (no drain) and drain groups. Relative survival was calculated as the ratio of observed to expected survival. Statistical comparison of the observed survival in the trial groups and the expected survival of respective population cohorts was performed with the one-sample log-rank test [1, 8]. An expected survival curve for the trial cohort was generated using the conditional (Ederer II) method [2]. Statistical significance was set at 5%.

## Results

**Fig. 1:**
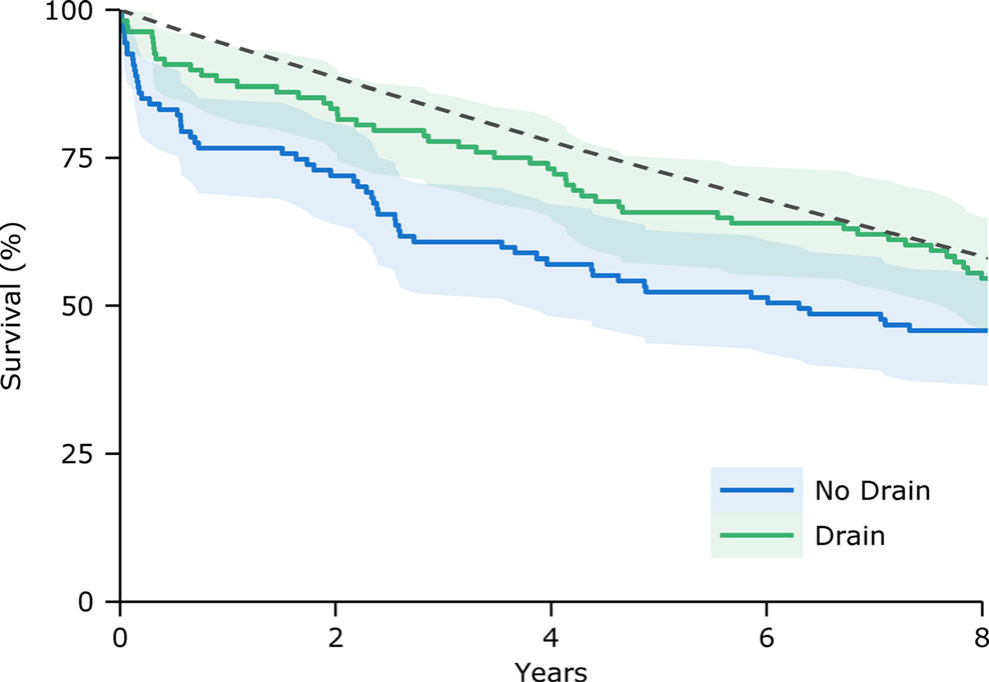
Kaplan-Meier survival curves for the trial groups (solid lines) with 95% confidence intervals (shaded regions) and the expected survival of a cohort of the general population matched for age and sex to the patients in the study (dashed line)

Patients in the trial had median age of 78 years (range 35–95) and 74.4% were male. At baseline the groups were well balanced [6]. At 5 years post-operatively 34.2% of patients in the drain group and 47.7% in the control group had died; comparison of 5-year survival curves showed a significant reduction in mortality associated with receiving a drain (χ^2^ = 4.9, p = 0.027; Fig 1). There was no significant difference when comparing the survival curves over 8 years (the time up to which complete follow-up was possible) or 10 years from surgery.

We then determined the relative survival of the trial groups against comparable age- and sex- matched cohorts of the general population. At 5 years the relative survival in the no drain group was 77.6% compared with 89.8% in the drain group. Comparison of the observed survival curves with expected survival (Fig. 1) found no significant difference in the drain group (χ^2^ = 2.8, p = 0.09) but patients who were not treated with a drain had significantly lower survival than the general population (χ^2^ = 11.8, p = 0.0006).

**Fig. 2:**
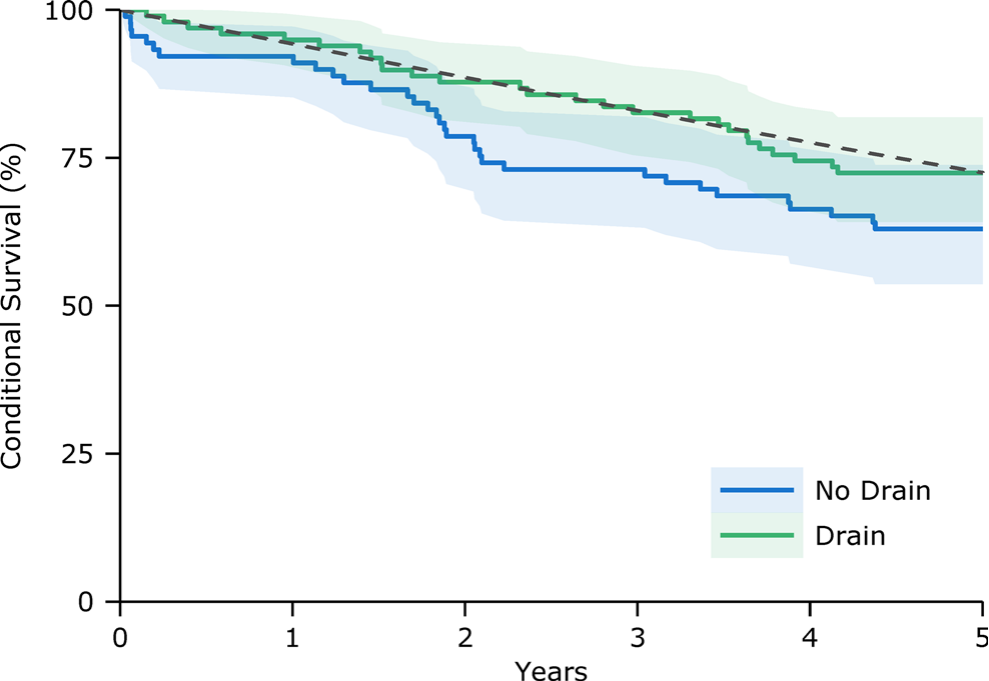
Conditional survival curves for patients who were alive at 6 months post-operatively (n = 187; solid lines) with 95% confidence intervals (shaded regions) and the expected survival of the matched general population (dashed line)

To exclude the potential bias of post-operative deaths within 6 months of surgery on the analysis of long-term relative survival the subset of trial patients alive at 6 months (n = 187; 98 in the drain group) was compared with the general population. Five-year relative survival, conditional on being alive at 6 months (Fig. 2), was again significantly lower in the control patients (χ^2^ = 4.9, p = 0.026) but not for the drain group (χ^2^ = 0.02, p = 0.897).

## Discussion

This study builds on the previously reported 6-month outcomes of a randomised controlled trial of subdural drains following burrhole drainage of CSDH [7]. Patients managed with a drain had significantly lower mortality at 6 months post-operatively and the present analysis confirms that a significant survival advantage remains at 5 years following surgery. Moreover, patients who received a drain had long-term survival that was not significantly different from that expected of the general population. In contrast, patients who did not receive a drain had significantly worse survival than expected. Importantly, this was not simply an effect of the higher early mortality in the control group: patients without a drain who survived at least 6 months continued to demonstrate lower relative survival, whereas the conditional survival in the drain group essentially followed that of the general population.

Subdural drains are effective in reducing the incidence of residual or recurrent haematoma that require reoperation [7]. By the same mechanism drains presumably also minimise subclinical recollections that contribute to slower neurological recovery and rehabilitation. Earlier mobilisation and faster discharge from hospital avoid the complications of prolonged inpatient admission and can have a long-term impact on whether patients regain functional independence. It is well recognised that reduced performance status and inability to do activities of daily living are associated with reduced life expectancy, particularly amongst the elderly [3].

Comparing the observed survival of the trial groups to that of the general population provides a valuable illustration of the efficacy of subdural drains over and above direct comparison of the trial groups. Particularly when trial participants are of a demographic with higher baseline mortality, calculating relative survival is a powerful method to detect excess deaths attributable to a disease or intervention, an inference that would be challenging to draw simply from inspecting survival curves alone [6].

Limitations of this study include the lack of later functional outcome assessments; it is not possible to know if the additional patients surviving in the subdural drain group also had a quality of life similar to the general population. Despite appearing to remain essentially parallel, comparisons of group survival curves beyond 5 years were not significant, reflecting inherent limits to the statistical power of the sample size in detecting differences as the number of patients at risk decreased during later follow-up. Similarly, the absence of a difference between the observed and expected survival of the drain group in the present analysis does not preclude that a larger study might resolve a statistically significant excess mortality.

Overall, the present findings underline the benefits of subdural drains following CSDH evacuation: patients managed with a drain survive longer and appear to have life expectancy similar to that of the general population. This evidence endorses the recommendation that standard practice for all burrhole evacuations of CSDH procedures should include, when safe, routine insertion of a subdural drain.
